# Infection and Vertical Transmission of Kamiti River Virus in Laboratory Bred *Aedes aegypti* Mosquitoes

**DOI:** 10.1673/031.007.5501

**Published:** 2007-11-06

**Authors:** Joel J. L. Lutomiah, Charles Mwandawiro, Japhet Magambo, Rosemary C. Sang

**Affiliations:** ^1^ Kenya Medical Research Institute (KEMRI), P.O. Box 54628-00200, Nairobi, Kenya.; ^2^ Jomo Kenyatta University of Agriculture and Technology (JKUAT), P.O. Box 62000-00200, Nairobi, Kenya

**Keywords:** Flavivirus, Flaviviridae

## Abstract

Kamiti river virus (KRV) is an insect-only *Flavivirus* that was isolated from field-collected *Ae.  macintoshi* mosquitoes in 1999, and is closely related to cell fusing agent virus. Both of these viruses belong to the family *Flaviviridae*, which also contains other viruses of medical importance, such as yellow fever virus, West Nile virus and dengue. Because *Ae. macintoshi* is the only known natural host to KRV, the main objective of this study was to establish the possibility that other mosquito hosts of the virus exist, by determining its ability to infect *Ae. aegypti* mosquitoes under laboratory conditions. The study also sought to determine the rates of infection and, subsequently, vertical transmission as a possible means of its maintenance and propagation in nature, given that it neither grows in vertebrate cells or mice. The mosquitoes were infected by the virus either as larvae or adults. Virus assay was done by re-isolation in tissue culture and indirect immunofluoresce assay methods. KRV infected *Ae. aegypti* mosquitoes, with the observed rates as high as 74 to 96 %. The virus was also transmitted vertically in these mosquitoes. Vertical transmission rates of 3.90 % were observed for the 2^nd^ and 3^rd^ ovarian cycles combined. These results suggest that *Ae. aegypti* mosquitoes are likely to be infected with KRV in nature, and that vertical transmission is the natural means by which it is maintained and propagated in this host, and possibly others.

## Introduction

Kamiti river virus (KRV) is an insect-only *Flavivirus* that was isolated from field-collected *Ae. macintoshi* mosquitoes that were collected as larvae from a flooded dambo on the shores of Kamiti river in the Ruiru division of Central Province, Kenya in 1999 (Sang et al. 2003). Assays by reverse-transcriptase polymerase chain reaction, and protein alignment defined KRV as being closely related to cell fusing agent virus which had been, until recently, the only known insect-only *Flavivirus* (Stollar and Thomas 1975). KRV is however the first insect-only *Flavivirus* to be isolated in nature as the cell fusing agent virus was isolated from laboratory-cultured *Ae. aegypti* mosquito cells (Stollar and Thomas 1975).

Extrinsic factors such as temperature, larval crowding, nutrition, and intrinsic factors such as genetic susceptibility, host preference and age of the adult mosquito affect the infection rates of viruses in mosquitoes (Baqar et al. 1980). Specific laboratory studies have shown that *Ae. aegypti* mosquitoes transmit the dengue-2 virus only if the mosquitoes are maintained at 30 °C, and the extrinsic incubation period is shortened if the extrinsic incubation temperature is increased to between 32 and 35 °C (Watts et al. 1987). However the temperature required to maintain maximum vector efficiency is dependent on the specific virus/vector system. Susceptibility of mosquitoes to infection by a virus also increases if freshly grown virus is used in the artificial infective meals (Miller 1987), as opposed to frozen stock. However, infection rates from nonfrozen virus in an artificial blood meal are significantly lower than those from the same dose ingested from viremic hamsters (Turell 1988).

Vertical transmission is the transmission of a virus from a parent host vector to the offspring through the female ova. It was demonstrated for LaCrosse virus in experimentally infected *Aedes triseriatus* (Watts *et al*.1973), and for Japanese encephalitis virus in *Culex. tritaeniorhynchus, Cx. annulus, Cx. quinquefasciatus*, and *Armigeres subalbatus* (Rosen et al. 1989) and, thereafter, for other viruses in a range of mosquito species. It involves virus entry into a fully formed egg during oviposition (Tesh and Cornet 1981), or venereally, though rarely, when an infected male mosquito passes over the virus to the offspring *via* the sperm during copulation with uninfected female (Simmons et al. 1931). It was experimentally demonstrated in the progeny of orally infected *Ae. aegypti* mosquitoes with regard to the yellow fever virus (Diallo et al. 2000) and Miller et al. (2000) demonstrated vertical transmission of West Nile virus in *Cx. univittatus* complex mosquitoes. It is considered to be a primary means by which some arboviruses are maintained during adverse environmental conditions when their arthropod hosts are either inactive or unable to survive. It is especially important in species found in cooler climates that are incapable of overwintering as adults, and is also a mechanism for virus persistence in environments where amplifying hosts are temporarily absent or immune (Miller et al. 2000).

High extrinsic incubation temperature is known to affect vertical transmission rates. In one study involving St. Louis encephalitis virus, infection rates were found to be much higher in F1 adult *Ae. epactius* mosquitoes reared at 18 °C than in those reared at 27 °C (Hardy et al. 1980). Vertical transmission rates decrease with successive ovarian cycles of infected mosquitoes (Beaty et al. 1980). This is a very rare occurrence in the first ovarian cycle progeny, it is mostly observed in the 2^nd^ and 3^rd^ ovarian cycles, with the rates being higher in the third cycle. The first ovarian cycle occurs before infection of ovaries while for the second ovarian cycle the ovaries are not as fully infected as they are in the third cycle due to the longer extrinsic incubation period (Diallo et al. 2000). Later, the rates decrease with the age of the mosquito as the ability to transmit the virus decreases with time (Scott et al. 1994).

Given that KRV was isolated from field-collected *Ae. macitoshi* alone, and has no known vertebrate host, this study sought to determine the susceptibility of *Ae. aegypti* mosquitoes to the virus, which would then suggest the likelihood that other mosquito hosts to the KRV exist in nature. Establishment of vertical transmission would mean that it is possibly the mechanism by which it is maintained among these hosts in nature.

## Materials and Methods

### Cell lines

Since KRV does not grow in vertebrate (Vero) cells, we used C6/36 cells, which are derived from *Ae. albopictus* mosquitoes. These cells are currently available in the Viral Hemorrhagic Fever Laboratory at the Kenya Medical Research Institute (KEMRI), Nairobi Kenya.

### Media

The C6/36 cells were grown in growth medium (Dulbecco's modified Eagle's medium) containing 5 % heat-inactivated fetal bovine serum, 2 % L-glutamine and 1 % antibiotic/antimycotic solution (with 10,000 units penicillin, 10 mg streptomycin and 25μg amphotericin B per ml). Infected cells were held in maintenance medium (Dulbecco's modified Eagle's medium) in which the fetal bovine serum had been lowered to 2%.

### Virus and liters

The SR-75 strain of KRV, which was used in this study, was isolated from adult *Ae. macintoshi* mosquitoes collected in the Sukari range. This virus, which had been preserved as a stock at the Viral Hemorrhagic Fever Laboratory at -80 °C and whose previous passage history was 4, was amplified by passaging twice in C6/36 cells to prepare stock virus for use in this study. Two 75-cm^2^ flasks of confluent C6/36 cells were infected with 600 μl of the virus suspension each and incubated for adsorption for 1 hr at 28 °C. The infected cells were thereafter maintained in 20 ml maintenance medium and incubated at 28 °C. Eight days post-infection the virus was harvested by scraping the cells from the flask surface and centrifuging the cell-virus-medium suspension at 1500 rpm for 15 minutes to remove cell debris. The supernatant containing the virus was stored in 0.5 ml aliquots at -80 °C. This was the source of the virus for infectious blood meals, and which was also used as the immunogen for the production of antiserum.

Virus titers were determined by titrating KRV in C6/36 cells. Ten-fold serial dilutions of the virus were made and 50 μl of each dilution was dispensed in duplicate wells of 96 well tissue culture plates. Each well was then seeded with 150 μl of C6/36 cell suspension containing approximately 10^6^ cells per ml, and incubated at 28 °C in a humidified chamber until development of a cytopathic effect. The viral titer was calculated using Karber formula (1931) to determine the log_10_TCID_50_ of KRV.

## Production of antiserum

Ten adult female NIH Swiss mice were immunized intraperitoneally with 0.3 ml of SR-75 infected tissue culture fluid mixed with complete Freunds adjuvant emulsion at a ratio of 1:1 on day 1. On day 14 the mice received the same treatment as above but using incomplete Freunds adjuvant. On day 21 the mice were inoculated with 0.3 ml of immunogen alone, which was followed by o.1 ml of Sarcoma cell suspension on day 28 and finally 0.3 ml of immunogen on day 35. The hyperimmunized mice were bled by heart puncture ten days following the last immunization. Blood was allowed to clot at 4 °C overnight and centrifuged at 700 rpm for 10 min to remove cells. The serum was collected and heat-inactivated at 56 °C for 30 min. It was then adsorbed with C6/36 cells to reduce non-specific binding; C6/36 cells from a 150-cm^2^ flask were washed in PBS and incubated with undiluted antiserum at room temperature in a centrifuge tube for 1.5 hr with constant mixing using a Pasteur pipette. Following adsorption, cells were removed by centrifugation at 1500 rpm for 10 min and the process repeated once. The antisera were then stored in appropriate amounts at -20 °C, until used.

### Mosquitoes and rearing

The F3 generation of the *Ae. aegypti* mosquitoes used were collected from Huruma village (co-ordinate readings: 257750–259250 Easting and 9863011–9864511 Northing), near Karura forest in Nairobi, Kenya. These mosquitoes were reared to F_3_ in the insectary maintained at approximately 28 °C and 75–80 % RH with a photoperiod of 16:8 L:D. The larvae were reared in white enamel pans and fed on Tetramin® fish food and liver powder. Adult mosquitoes were continuously given 10 % sucrose solution as a carbohydrate source and fed on normal laboratory mice to stimulate egg production.

## Mosquito infection

Mosquitoes were orally exposed to the virus either as adults (adult infection), or as larvae (larval infection).

### Adult infection

One hundred and fifty 3-day-old adult mosquitoes held in 4-liter plastic cages were starved of sucrose and deprived of water for 24 hours prior to blood feeding. The mosquitoes were orally exposed to an infectious blood meal prepared by mixing a 1:1 ratio of the stock virus suspension with defibrinated rabbit blood. The blood meal was placed in membrane feeders covered with mouse skin. Blood meals were maintained at a constant temperature of 37 °C throughout the 45 minutes feeding period. Fully engorged mosquitoes were selected with an aspirator and held at a constant temperature of 28 °C and 70–80 % RH, during a 16:8 L:D, and provided with a 10 % sucrose solution in cotton wicks for an extrinsic incubation period of 14 days. Sampled mosquitoes were killed by chilling at -70 °C before being used for the virus assay. Samples of the infectious blood meal were taken before and after the feeding period and immediately titrated in C6/36 cells to determine infecting virus titer. In all mosquito infections, infective blood meals of virus titer ranging from 3.0 × 10^7^ to 3.2 × 10^7^ per ml were used, with no significant change over the feeding period.

### Larval infection

Second instar larvae of mosquitoes were exposed to KRV-infected cells eight days after infection at 28 °C in 25-cm^2^ flasks, and left at the same temperature for 30 minutes for maximum feeding. The larvae were then removed using a Pasteur pipette, thoroughly rinsed in distilled water and transferred to normal rearing conditions in the insectary. The virus assay was performed on individual mosquitoes 14 days after emergence.

### Vertical transmission rates

*Ae. aegypti* mosquitoes orally exposed to KRV were subjected to single-pair mating experiments. A virgin male and female mosquito (20 pairs) were placed together in individual cylindrical (20 × 20 cm) cardboard containers, covered with a fine nylon mesh at the top. Each container had a 5 × 4 cm plastic jar partially filled with water and lined with brown paper for oviposition. 3 to 5 days later, the male mosquito was aspirated from the containers and the female was fed on a mouse. After oviposition, the eggs were hatched and the offspring reared separately. Individual parent mosquitoes were then assayed for KRV infection. Only F_1_ offspring of the parent mosquitoes that tested positive for KRV were tested for KRV-infection as adults by isolation in tissue culture.

### Virus assay: Isolation in tissue culture

Mosquitoes were selected 14 days after virus exposure and sacrificed by chilling. Individual mosquitoes were then placed in chilled Eppendorf vials and triturated in 1 ml growth medium using sterile pestles. Mosquito suspensions were clarified by centrifugation at 3,000 rpm (Mini Centrifuge, C-1200, www.labnetlink.com) at 4 °C for 1 min. 50 μl of each suspension was inoculated onto 96-well, flat-bottom-cell culture plates (50 μl per well) and seeded with 150 μl of C6/36 cell suspension containing 10^6^ cells per ml. The plates were incubated at 28 °C and cells monitored daily for development of characteristic cytopathic effects. KRV-infected and non-infected C6/36 cells incubated for the same period of time were included as negative and positive controls respectively.

**Table 1.**
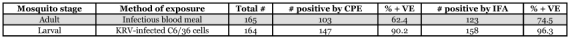
Infection rates of KRV in *Aedes aegypti* mosquitoes. The number and percentage of samples of infected female mosquitoes assayed for viral infection by both isolation in tissue culture and IFA methods.

### Virus assay: Indirect immunofluorescence assay (IFA)

Six days after infection, virus-infected cells were gently scraped from the well surface and aspirated from each well using a Pasteur pipette. The cells were washed once in 1 × phosphate buffered saline (PBS) and separated by centrifugation at 2,000 rpm (Kubota, KS-5000) at room temperature for 10 minutes. A PBS-cell suspension was prepared and 20 μl of it spotted on each well of the 12-well Teflon coated slides and air-dried. The cells were fixed in cold acetone at -20 °C for 30 minutes and stained with KRV antiserum diluted 1:20 in PBS. The slides were incubated for 1 hr at 37 °C in a humidified chamber and then washed three times in PBS for 5 min each. 20 μl of a commercial anti-mouse Immunoglobin G (IgG) conjugated with fluorescein-isothiocyanate (FITC), diluted 1:32 in PBS, was added to each well. Evans blue was also added to the conjugate at the ratio of 1:10. The slides were again incubated for 1 hr at 37 °C in a humidified chamber and washed as above in the dark and mounted with cover slips using mounting solution consisting of Mcllvaine's Buffer: Glycerol (M-7534) in a ratio of 1:1. The slides were examined under an Olympus BH-2 epifluorescence microscope equipped with a 20x objective lens, an HBO lOO-W high-pressure mercury burner, and an IF-49O exciter filter.

## Results

### Infection rates

About 62 % of adult female mosquitoes orally exposed to infectious blood meals of virus titer range 3.02 × 10^7^ to 3.18 × 10^7^ per milliliter, tested positive for KRV by isolation in tissue culture, while 74.5 % infections were detected by IFA. There was no significant change in the KRV titers over the feeding period. For those females exposed to KRV-infected cells as larvae, 90.2% infections were detected by isolation in tissue culture and 96.3 % by IFA (Table 1). In both cases, there were significant differences between the number of KRV-positive mosquitoes detected by isolation in tissue culture and IFA (*p* = 0.028 for those exposed as adults and *p* = 0.012 for those exposed as larvae). There was also a statistically significant difference (*p* = 0.039) in the rates of infection between mosquitoes exposed as adults and those exposed as larvae, by IFA. However the difference was not significant (*p* = 0.056) by isolation in tissue culture.

### Vertical transmission rates

Vertical transmission of KRV in orally infected laboratory-colonized *Ae. aegypti* female mosquitoes to their progeny was experimentally demonstrated by isolation in tissue culture. Infected progeny were detected in the F1 generation from the combined 2^nd^ and 3^rd^ ovarian cycles of the 13 parent mosquitoes that had been found to be infected with KRV. Total filial infection rate for the two cycles was 14:410, representing 3.90 %. The rates were also higher in females (4.53 96) than in males (2.76 %) (Table 2), although this difference was not significant, *p*= 0.076.

**Table 2.**
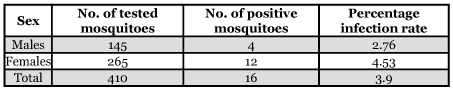
Vertical transmission rates. The number and percentage of samples of emerging mosquitoes assayed for viral infection by isolation in tissue culture.

## Discussion

High infection rates (74 and 96 %) of KRV in *Ae. aegypti* mosquitoes orally exposed to KRV are reported using laboratory-reared mosquitoes. These observations suggest that *Ae. aegypti* mosquitoes are likely to be infected with KRV in nature. This hypothesis is supported by previous isolation of KRV-like virus from *Ae. aegypti* mosquitoes (RC Sang, unpublished data). However, cross-sectional field studies are needed to confirm this, as well as if other species of mosquitoes are likely to be infected with KRV or related viruses in nature.

Higher infection rates (90.2 and 96.3%) were observed in mosquitoes exposed as larvae. This is in agreement with expectation, as in the adult female the blood meal initiates the formation of a peritrophic membrane within mosquitoes (Miller and Lehane 1993; Billingsley 1990) that physically separates it from the midgut epithelium. Since viruses that fail to exit the blood bolus within a few hours become trapped by the developing peritrophic matrix (Whitfield et al. 1973), those that exit to access receptor sites and effect infection are fewer than those present in the midgut. In contrast, consumption of KRV-infected cells by larvae results in larvae individually ingesting more viral particles than those ingested by individual adult mosquito through an infectious blood meal. Since mosquito cells have fewer receptors for Venezuelan equine encephalitis virus (Ludwig et al. 1996), if this is also true for KRV receptors increased viral particles may optimize chances of the virus coming in contact with cell receptors thus enhancing infection and, subsequently, infection rates.

Ingested viruses are also exposed to a hash midgut environment that include an abrupt change in temperature and pH, the onset of blood digestion by proteolytic enzymes and the loss of fluid nature of the ingested blood (Beernsten et al. 2000), which may affect the pathogen (Gass 1977; Shahabuddin 1996) and influence infection rates. Since these factors may be initiated by taking the blood meal, or occur within the bloodmeal, their effect may be less apparent in larvae that do not consume a blood meal. The high larval infection rates may also have been due to the freshly grown virus that is known to increase susceptibility of *Ae. aegypti* to infection (Miller 1987). It has also been observed that the mid-guts of *Ae. aegypti* females have pH range of 7.40–7.52 (Corena et al. 2005), which is lower than that of larval mid-guts pH range 9.5–11 (Corena et al. 2002). Although not supported by the necessary data, the possibility that mid-gut pH has an effect on the viability of the virus, and that a more alkaline environment is favorable, cannot be discounted. This larval infection data suggests that using infected C6/36 cells may have caused a bias in the results on infection rate. Although the virus for making infectious culture and blood meal came from the same stock, the fact that the virus titer in individual cells was not determined could also have contributed to the bias.

The higher infection rates (>74 %) obtained by IFA is indicative of the fact that this method is more sensitive compared to that of virus isolation in tissue culture. The lower rates detected by the latter method do not therefore rule out the possibility that some mosquitoes that tested negative were actually infected at the time of assay but the viremic level may have been too low to cause detectable cytopathic effects in the tissue culture, yet high enough to be detected by IFA. The difference in infection rates observed between the two methods was significant, *p*= 0.028.

We have observed that orally infected *Ae. aegypti* mosquitoes can vertically transmit KRV to their progeny. The high overall first filial infection rates (3.41 %) were attributed to the fact that KRV is an insect-only Flavivirus with enhanced infection. There was no statistically significant difference between vertical transmission rates in females *vs.* those in males, *p*= 0.076, although the rate was twice as high in females. Significant differences in infection rates between male and females were found by Diallo et al. 2000 with regard to yellow fever virus and *Ae. aegypti* mosquitoes. These investigators explained that the higher rates in females than males was due to venereal transmission of the virus to non-infected females by infected males during mating.

Although our study did not specifically demonstrate venereal transmission, it is a process that may increase the prevalence of this virus in nature. As Gaunt et al. 2001 pointed out, the evolution and dispersal patterns of Flaviviruses have been determined through a combination of constraints imposed by the arthropod vector and the associated ecology. Various laboratory experiments have shown that vertical transmission rates tend to vary widely between different mosquito species and viruses. Vertical transmission rates as low as 0.17 % for yellow fever virus in *Ae. aegypti* (Beaty et al. 1980) have been reported in some studies. Others have reported higher rates than those observed in our study. Freier and Rosen (1987), demonstrated that *Ae. cooki* mosquitoes transmitted Dengue 1 and 3 viruses to 6.7 % of their offspring, while 4.6 % of filial infection was observed in *Ae. polynesiensis* for the same virus.

Previous studies with KRV showed that it is an insect-only Flavivirus most closely related to the cell fusing agent virus. We have observed that *Ae. aegypti* mosquitoes are highly susceptible to KRV, and that infected mosquitoes are able to vertically transmit the virus to their progeny. This is the likely mechanism by which the virus is maintained and propagated among its natural hosts. These observations contribute to the existing knowledge about KRV. Studies should be carried out to determine the susceptibility of other mosquito species to KRV, and to compare the filial infection rates of mosquitoes infected as adults and those infected as larvae.

## Abbreviations

IFA -: Indirect immunofluoresce assay
KRV -: Kamiti river virus
